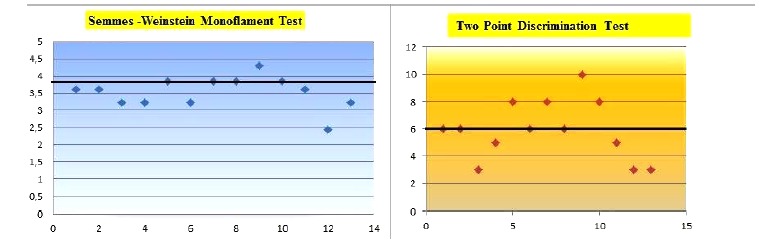# Lateral Antebrachial Cutaneous Nerve as a Donor Source for Digital Nerve Grafting: A Concept Revisited

**DOI:** 10.2174/1874325001711011041

**Published:** 2017-08-29

**Authors:** Mehmet Bekir Unal, Kemal Gokkus, Evrim Sirin, Eren Cansü

**Affiliations:** 1 Department of orthopaedic surgery Göztepe Medicalpark Hospital, Merdivenköy, 23 Nisan Sok. No:17, 34732 Kadıköy/Istanbul, Turkey; 2 Department of orthopaedic surgery Attending Surgeon Memorial Antalya Hospital, Zafer Mah.Yildirim Beyazit Cad. Number:91 Kepez /Antalya/Turkey; 3 Department of orthopaedic surgery Fatih Sultan Mehmet Research and Education Hospital . E5 Karayolu Üzeri İçerenköy, 34752 Ataşehir /Istanbul, Turkey; 4 Marmara University Faculty of Medicine, Pendik Research and Education Hospital. Department of Orthopaedics and Traumatology, Fevzi Çakmak Mah., Mimar Sinan Caddesi No:10, 34899 Pendik/İstanbul, Turkey

**Keywords:** Digital nerve injury, Nerve grafting, Lateral antebrachial, Cutaneous nerve (LACN), Donor source, Autografting

## Abstract

**Objective:**

The main objective of this study is to evaluate the availability of lateral antebrachial cutaneous nerve (LACN) autograft for acute or delayed repair of segmented digital nerve injuries.

**Patients and Methods:**

13 digital nerve defects of 11 patients; treated with interposition of LACN graft that harvested from ipsilateral extremity were included in the study. Mean follow up period was 35, 7 months. The mean time from injury to grafting is 53, 3 days. The results of the mean 2PDT and SWMT values of injured /uninjured finger at the end of follow up period were evaluated with Paired T test. The correlation between the defect length and the difference of 2PDT, SWMT values between the uninjured and injured finger at the end of follow up period; were evaluated with Pearson - correlation analysis.

**Results:**

The mean value of our 2PDT and SWMT results are ~5,923, ~3, 52, respectively in which can be interpreted between the normal and diminished light touch. The defect length and difference percentage of SWMT values is positively and significantly correlated statistically. Mean length of interposed nerve grafts was 18.5 mm. The age of the patient and the mean values of 2PDT and SWMT with the difference % of 2PDT and % of SWMT are not statistically correlated.

**Conclusion:**

Based on results regarding sensory regaining at recipient side and negligible sensory deficit at harvesting side, we suggest that lateral antebrachial cutaneous nerve might be a valuable graft option for digital nerve defects.

## 1 INTRODUCTION

Injury to digital nerves of the hand is common and they are the most frequent cause of sensory impairment of the hand [1]. A nerve gap is defined as “the distance between two ends of a divided nerve”. It is not only caused by the nerve tissue lost due to trauma or debridement, but also by actual retraction of nerve stumps [2].

Peripheral nerve injuries with gaps larger than 1-2 cm require bridging strategies for nerve repair/coaptation. There are various reconstruction methods for such significant nerve gaps such as autologous nerve grafts, interposition of venous or arterial segments or interpositions of synthetic conduits [2, 3].

Achievement of satisfactory functional recovery following the repair of a segmental peripheral nerve defect always posed a challenge for the surgeons. It has long been established that the repair of a segmental defect of nerve usually gives very poor results when the nerve coaptation of stumps is done under tension [4-7].

Using autologous nerve grafts for nerve defect bridging is a relatively common procedure employed in hand surgery. In such cases, interposition autologous nerve graft is considered as the gold standard of treatment and is associated with the best outcomes [8].

Even though digital nerve defects are commonly encountered in hand injuries, there are a very few number of reports in the literature regarding lateral antebrachial cutaneous nerve (LACN) as a donor source. The main objective of this study is to evaluate the availability of lateral antebrachial cutaneous nerve (LACN) autograft for acute, segmented or delayed repair of digital nerve injuries in a clinical trial.

## 2 MATERIALS AND METHODS

### 2.1 Patients

 13 digital nerve defects of 11 patients (9 males, 2 female); treated with interposition of lateral antebrachial cutaneous nerve graft that harvested from ipsilateral extremity over a 5-year period between 2007 and 2012 were included in the study. The age, gender, the follow up period, the existence of concomitant injury, the defect length (gap distance), site of injury were documented (Table **1**). Mean age of patients was 27 (17-38). Mean follow up period was 35, 7 months. The defect length (gap distance -mean size of interposed nerve grafts) was 18.5 (15-25) mm. The mean time from injury to grafting was 53, 3 days. The mechanism of injury were the same for all the cases. (Sharp objects injured all patients)

### 2.2 Surgical Technique

**Fig. (1):**
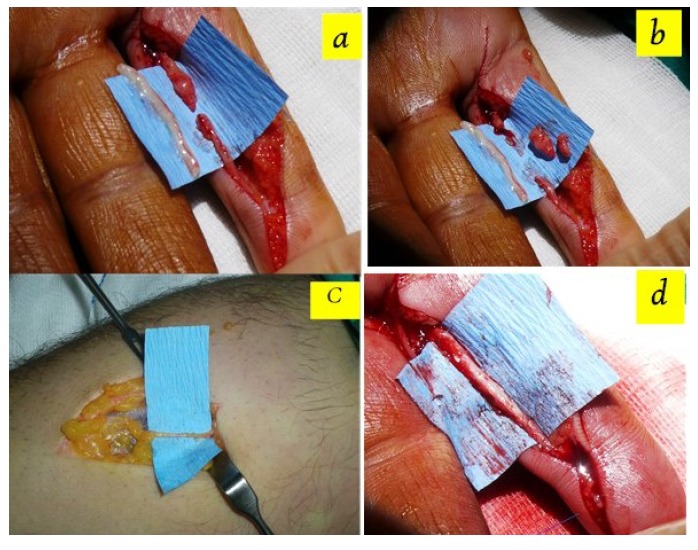
Notice the neuroma formation at upper stump of the injury site **b .** Notice the excised neuroma, the clean stumps was prepared. **c.** The appearance of lateral antebrachial cutaneous nerve in donor field **d.** The appearance of repaired digital nerve after autografting with lateral antebrachial cutaneous nerve.

Patients were operated under pneumatic tourniquets inflated to 250 mmHg. Appropriate magnification during the operation was obtained using operation field microscopy. Previous scar lines were extended using zigzag incisions and neurovascular bundles were exposed in patients with chronic injury. Neuroma formations at nerve stumps were resected to expose healthy nerve fascicules (Figs. **1a**, **b**). In acute cases, nerve ends were dissected and were prepared for repair. Following the nerve gap measurement, graft harvesting was initiated. Initially, biceps tendon insertion was palpated at cubital fossa in the forearm in full supine position and a longitudinal incision was done, just lateral to the longitudinal axis of biceps tendon.

Cephalic vein was identified within one cm to the radial side of incision following gentle retraction of subcutaneous fat. Nerves were continuously identified along the course of cephalic vein with ramifying branches in some parts. Nerve graft, a few millimeters larger than the defect, was harvested from its anatomical site (Fig. **1c**). Proximal nerve ends were cauterized and buried under forearm fascia. Skin closure was done using interrupted sutures. Mean size of interposed nerve grafts was 18.5 (15-25) mm. All nerve repairs were performed in Zone II. Epineural neuroraphy was performed using 9/0 nylon sutures. (Fig. **1d**) Evaluation of procedure-related morbidities was done with percussion throughout the donor and recipient nerve tracts in order to check for Tinnel’s sign, which indicated a neuroma formation. The patients were also examined for sensation disturbances in forearm region, which innervated with harvested lateral antebrachial cutaneous nerve branch and for presence of a disabling scar formation. The return of sensation distal to neuroraphy was evaluated using 2PDT and SWMFT at 3 rd. month and end of follow up period.

### 2.3 Data Collection

The SWMT was performed with Touch-Test ™ 20 Piece Full Kit in accordance with instructions of the instrument [9].

Static -2PDT was performed with Touch-Test ® two point discriminator apparatus in accordance with instructions of the instrument [10].

### 2.4 Statistical Method

The mean values of 2PDT and SWMT (at the end of follow up period) were analyzed with Paired T test and the results were showed in table (Table **2**).

The correlation between the defect length and the % difference of 2PDT and SWMT values between the uninjured parallel finger and injured finger at the end of follow up period were evaluated with Pearson correlation analysis. The results interpreted with correlation coefficient [11] in which we can define how strong connection between two variables. (Rxy=correlation coefficient, 0.1 <r_xy_ <0.3: weak, 0.3 <r_xy_ <0.5: moderate, 0.5 <r_xy_ <0.7: visible, 0.7 <r_xy_ <0.9: high, 0.9 <r_xy_ <1: very high).

## 3 RESULTS

### 3.1 Donor Side Results

5 patients were diagnosed with hypoesthesia in a small area and 6 patients had positive Tinel’s sign with only strong percussion at the donor field. The patients were not concerned about the area of numbness.

### 3.2 Recipient Side Results

No Tinel’s sign was detected on neuroraphy sites when recipient fields were examined. The mean value of 2PDT and SWMT of injured finger is significantly higher than (worse than) the uninjured finger. (p=0, 0001). [See the Table **2**]. The defect length and the difference percentage of 2PDT values values (injured finger) is not statistically significantly correlated (p>0, 05) (Table **3**). The defect length and difference percentage of SWMT values is positively statistically significant correlated (r=0,557 p=0,048) [Table **3**].

### 3.3 Interpretation SWMT Results (Injured Recipient Side at Final Follow Up)

The values with ≤3, 84 constitutes the majority. The mean value of SWMT is ~3, 52 in which can be interpreted between the normal and diminished light touch [10] (Table **4**).

### 3.4 Interpretation of 2PDT Results (Injured Recipient Side at Final Follow Up)

2PDT values were between 3-5 mm in 5 nerves and 6-10 mm in 8 nerves. The distribution of the values with ≤6mm constitutes the majority (Table **4**). The mean value of our 2PD result is ~5,923 in which can be interpreted in normal limits [11].

### 3.5 Other Parameters

The mean time from injury to grafting and the mean values of 2PDT with the difference % of 2PDT, SWMT with the difference % of SWMT are not statistically significant correlated (p>0, 05) (Table **3**).

The age of the patient and the mean values of 2PDT with the difference % of 2PDT, SWMT with the difference % of SWMT are not statistically significant correlated (p>0, 05) (Table **3**)

### 3.6 Interpretation of the General Results

Despite the mean values of injured fingers are significantly higher than (worse than) the uninjured fingers regarding to these two tests, the majority of the fingers stands near the normal sensation.

## DISCUSSION

According to our study, using of lateral antebrachial cutaneous nerve (LACN) as a donor source for mean 18,5 mm - digital nerve defect, improved 2PDT and SWMT measurements after the surgery on mean follow up time 35, 7 months.

This study revealed that the increasing defect length worsens the SWMT and % difference SWMFT result. Differently from the previous studies defect length was found to be determinant factor on senstaional regaining in our study.

While the % difference 2PDT is not affected from increasing gap size. Small sample size of our study is suggested to be reason of this outcome with large interinduvudial variation feature of 2PDT.

The age and the time interval between injury and repair was not found to be effective factor on regaining sensorial function of the digit in our study.

The result regarding to age should not be generalized, because the patient sample of our study consisted young and middle aged patient group. So we could not evaluate the age older than 38 years old.

The relationship between the age of injury (time interval between injury and repair) and return of sensation were assessed in previous studies (McFarlane and Mayer’s study and Tenny *et al*’s study) [1, 12]. Both studies concluded that the age of injury (time interval between injury and repair) was not determinant factor on regaining sensorial function of the digit such as our study. This parallelism might be a contribution to scientific knowledge regarding digital nerve injuries.

There were a few previously published studies found in the literature that deals with LACN as a donor source.

The use of lateral antebrachial cutaneous nerve as a graft for digital nerve repair was initially reported by McFarlane and Mayer [12] (1976). In their study, the authors evaluated the availability of this autograft in a clinical setting using 13 grafts. They found out LACN was an excellent donor nerve since it is relatively easy to obtain, has a satisfactory/appropriate caliber for grafting and provides a sufficient length for digital nerve grafts. They performed 2PDT and nin-hydrin sweat test to assess return of sensation. The regained 2PDT values were also investigated, but their results were poorer than us in the terms of 2PD criteria. This can be explained by the mean time from injury to grafting and the mean defect sizes are higher than us in their cohort. In contrast with us, they did not seek any correlation between the defect length and values of post-operative regaining sensation. Parallel with us; the age and the time interval between injury and repair was not found to be determinant factor on return of sensation in their study.

Tenny *et al.* [1] (1984) presented a large cohort (42 studies) on LACN auto grafts. The authors reported better clinical results with lateral antebrachial cutaneous nerve (LACN). The evaluation of the patients was done using various testing methods such as sharp versus dull discrimination, heat sensitivity, vibration, dynamic and static two-point discrimination, perception test, texture differentiation and shape identification. They did not find any obvious relationship between the length of the graft or time- interval from injury to grafting and the results. However, they found correlation between the age and the amount of sensory return. Opposingly Tenny *et al*’s study, we could not find any relationship between the age and clinical results. Our group consist of young people, so lack of older age patients might cause these results. We could not compare our clinical results with this study, because in this study the scale of the British medical research council and other parameters were utilized.

Pilanci O *et al.* [13] presented a cohort (15 patients) that aimed to evaluate recipient and the donor site sensorial results of the lateral antebrachial cutaneous nerve (LACN) in digital nerve restoration. They found out that the restoration of digital nerve defects with LACN graft had satisfactory results. They performed this study on a homogenous patient group that consisted with chronic cases .This feature strengthened their study. In contrast with this study, our patient group were consisted with acute and chronic cases. If we closely examine two cohorts, in our group only two patients had acute injuries, and the other cases had chronic injuries with similar with Pilanci O *et al*’s group. Also the mean time interval from injury to grafting day were very similar in two groups .(Our study 53,3 days, Pilanci *et al*’s study 50, 7 days).

Chiu CK *et al* [14] emphasized the presence of trifurcation on LACN and availability for digital nerve grafting in a letter.

There is no single test, which clearly documents of nerve function, and therefore, we combined the most generally accepted methods. 2PDT is the most reliable index of return of sensation [12, 15]. The Semmes Weinstein monofilament has acceptable intra-rater & inter-rater reliability [16]. This test can detect change over time i.e. quality of neural return, progression, or deterioration [17, 18].

Therefore, these two assessment methods were fitted to our search. We used only 2PDT to assess return of sensation and we added SWMT to assess pressure needed to detect touch [16].

Apart from McFarlane and Mayer [^[^12^]^] Pilanci O [13] and Tenny *et al*’s [1] cohort study, there were no previous publications/studies regarding the clinical availability of LACN autografting at digital nerve defects.

Tank MS *et al* [19] did a histomorphometric evaluation of the fascicular pattern of LACN and digital nerves. They reported that the LACN closely resembled the original fascicular pattern of digital nerve and therefore, can be considered as a highly suitable donor for digital nerve grafts. Higgins *et al.* [8] performed a cadaver study based on microscopic feature of nerves. Their results said that the LACN was most appropriate for Zone 2 and 3 injuries.

Tenenhaus M *et al* [20] and some various authors reported the advantages of PIN as a donor source for digital nerve grafting from their experience and concluded that this nerve graft affords minimal donor-site morbidity; in addition to having an appropriate size to match for digital nerve grafts distal to proximal phalanx. [3, 20-22] According to the our point of view, to obtain PIN necessitates pronation of forearm. Thus during the surgery hand should stay at two position comparing with one position (supination).This can cause time and concentration loss.

Donor nerve selection is heavily dependent on harvesting ease and post-surgical morbidity rates. An ideal donor nerve should be easy to locate, surgically accessible, have sufficiently long segments without lateral branches with small overall diameters and well developed fascia. In addition, the sensory deficit caused by the harvesting should occur in a non-critical cutaneous region [8].

Under some special circumstances where autografting is not available (such as patient not consenting to autografting or a relatively short surgical period is necessary due to patient’s medical situation), nerve conduits can also be used as an alternative to autografting.

The main limitations of our study include the lack of detailed scoring systems such as texture differentiation, British Medical Council Scale, American Society for Surgery of the Hand (ASSH) guidelines and Disabilities of the Arm, Shoulder, and Hand (DASH) scores for assessment of functional recovery and small size of our cohort.

## CONCLUSION

Based on satisfactory results regarding to sensory regaining at recipient side and negligible sensory deficit and complaints at harvesting side, we suggest that lateral antebrachial cutaneous nerve might be a valuable graft option for digital nerve defects. Moreover, our study suggest that the increasing of the gap size between the nerve ends worsens the SWMT and percentage difference SWMFT results.

## Figures and Tables

**Table 1:** Comparative results (at the end of follow up period) of 2PDT and SWMT between normal parelel finger with inured side, gender, age, the mean time from injury to grafting, defect (gap) length, injury mechanisms, concomitant injury, follow up period.

Patient No	Group 1 Two-Point Discrimination Mm (injured finger)	**Group 2** Two-Point Discrimination mm (un injured side-parellel finger)	Group 3 The Semmes-Weinstein Monofilament Test(injured finger)	**Group 4** The Semmes-Weinstein Monofilament Test(un-injured Side parellel finger)	Group 5 The Mean Time from Injury to Grafting	Group 6 Defect length (mm)	Group 7 Length of Follow-up Period (Month)	Group 9 Concomitant Injury	Group 10 Age/sex
1	6 6	5 5	3,61 3,61	2,83 2,83	Same day	15/15	42	FDP tendon	26 m
2	3	3	3,22	2,83	4 months	20	40	FPL tendon	30 m
3	5	5	3,22	2,83	15 days	10	32	no	24 m
4	8	5	3,84	2,83	5 days	23	28	no	24 m
5	6	3	3,22	2,83	5 months	20	56	no	38 f
6	8 6	3 3	3,84 3,84	2,83 2,83	Same day	25/25	50	FPL tendon	35 m
7	10	5	4,31	3,61	6 months	20	25	no	28 f
8	8	6	3,84	2,83	10 days	22	34	no	22 m
9	5	3	3,61	2,83	4 months	18	32	no	28 m
10	3	3	2,44	2,83	2 days	13	30	no	17 m
11	3	3	3,22	2,83	3 months	15	24	no	25 m

**Table 2:** The mean value of two-point discrimination of injured finger is significantly higher than (worse than) the un-injured finger . (p=0,0001). The mean value of **The Semmes-Weinstein monofilament test** of injured finger finger is significantly higher than the un-injured finger . (p=0,0001).

	**Injured Finger**	**Un- Injured Side** **Same Finger**	**P**
Two-point discrimination (mm)	5,92±2,18	3,85±0,8	0,0001
The Semmes-Weinstein monofilament test	3,52±0,46	2,73±0,25	0,0001

Paired T-test.

**Table 3:** The defect length and the difference % of 2PDT values is not statiscially significant correlated (p>0,05). The defect length and the difference % of Semmes-Weinstein monofilament test is positively statiscially significant correlated (r=0,557 p=0,048). The mean time from injury to grafting and the mean values of 2PDT with the difference % of 2PDT, SWMT with the difference % of SWMT are not statiscially significant correlated (p>0,05). The age of the patient and the mean values of 2PDT with the difference % of 2PDT, SWMT with the difference % of SWMT are not statiscially significant correlated (p>0,05).

		**Defect Length**	**Time of Repair After Injury**	**Age**
Difference %2PDT(mm)	r	0,445	-0,324	0,407
	p	0,128	0,281	0,214
difference % SWMT	r	0,557	0,131	0,288
	p	0,048	0,67	0,391
The Semmes-Weinstein monofilament test (un injured finger)	r p	0,277 0,36	-0,497 0,084	0,215 0,525
The Semmes-Weinstein monofilament test (injured finger)	r p	0,608 0,027	-0,277 0,36	0,305 0,361
Two-point discrimination mm (injured finger)	r p	0,544 0,055	-0,425 0,148	0,236 0,485
Two-point discrimination mm (un injured finger)	r p	0,468 0,106	-0,4 0,176	0,021 0,952

Pearson Correlation Test

**Table 4:** Semmes-Weinstein monoflament test results in 13 patients. The distribution of the values with ≤3,84 constitutes the majority. Two point discrimination test results ,notice the distrubition of the patient , majority of the patient stands below 6 mm.